# Training Dental Researchers in Digital Outreach: A Course Model for Science Communication and Public Engagement

**DOI:** 10.1002/jdd.70137

**Published:** 2025-12-30

**Authors:** Vandilson Rodrigues

**Affiliations:** ^1^ School of Dentistry Federal University of Maranhão São Luís Maranhão Brazil

**Keywords:** dental education, science communication, social media

## 1 Problem

A significant barrier to effective science outreach among dental professionals is the lack of formal training in communication during academic education [1]. Traditional dental curricula prioritize clinical competencies and biomedical knowledge [2], often neglecting the development of skills in science communication, digital media production, and public engagement. As a result, many dental professionals can feel unprepared to share their research findings with non‐specialist audiences. This gap reduces the reach and impact of scientific advancements in dentistry and contributes to the persistence of misinformation regarding oral health.

## 2 Solution

Effective online outreach is important for dental researchers aiming to bridge the gap between scientific discovery and public understanding. In an era dominated by digital communication and social media, the ability to translate complex research findings into accessible, engaging, and accurate content has become an essential skill [3]. Disseminating evidence‐based information to non‐health professionals, including patients, educators, policy‐makers, and the general public, not only enhances the visibility and societal relevance of dental research but also combats misinformation and builds public trust in science [4]. By using clear language, compelling storytelling, and audiovisual tools tailored for diverse platforms, dental researchers can amplify the impact of their work, encourage informed decision‐making, and contribute to a more scientifically literate society [5].

To develop these competencies, a graduate‐level course was implemented at the Federal University of Maranhao, Brazil, to train dental researchers in effective strategies for research outreach and dissemination. This innovative course, designed specifically for masters and doctoral students, blends theory and practice in science communication, focusing on translating complex research into formats that are accessible and engaging for non‐specialist audiences. The course is structured into three comprehensive modules. The first module covers the fundamentals of digital communication and research metrics. The second focuses on storytelling, content scripting, and audience adaptation. The third provides practical training in audiovisual production and editing (Table 1). During the course, all students completed training activities that involved creating a graphical abstract, writing a news article for the institutional website, and producing a video abstract based on a preselected scientific article.

**TABLE 1:** Structure of the course in digital science communication: learning content and practical activities by module.

Module	Learning content	Practical activities
Module 1: Digital communication and research metrics	Principles of digital science communication Science popularization and public engagement Use of social media for research dissemination Traditional bibliometrics (e.g., impact factor, *h*‐index) Alternative metrics (e.g., Altmetrics, PlumX)	Creating and updating author profiles (ORCID, Scopus, Web of Science) Identifying and interpreting metrics at the journal, article, and author levels
Module 2: Planning audiovisual resources	Audience analysis and segmentation Practical implications of dental research Characteristics of science news writing Key elements of effective graphical and video abstracts	Selecting a scientific article for outreach Synthesizing and highlighting main findings Identifying target audiences Structuring key messages and study relevance Adapting language and style for audiovisual formats Writing scripts for video abstracts Selecting images and scenes for production
Module 3: Audiovisual production and editing	Tools and techniques for image and video production Basic editing principles for scientific communication	Presenting initial drafts of science news, graphical abstracts, and video abstracts Peer feedback and group discussion Revising and finalizing multimedia materials

## 3 Results

**FIGURE 1:**
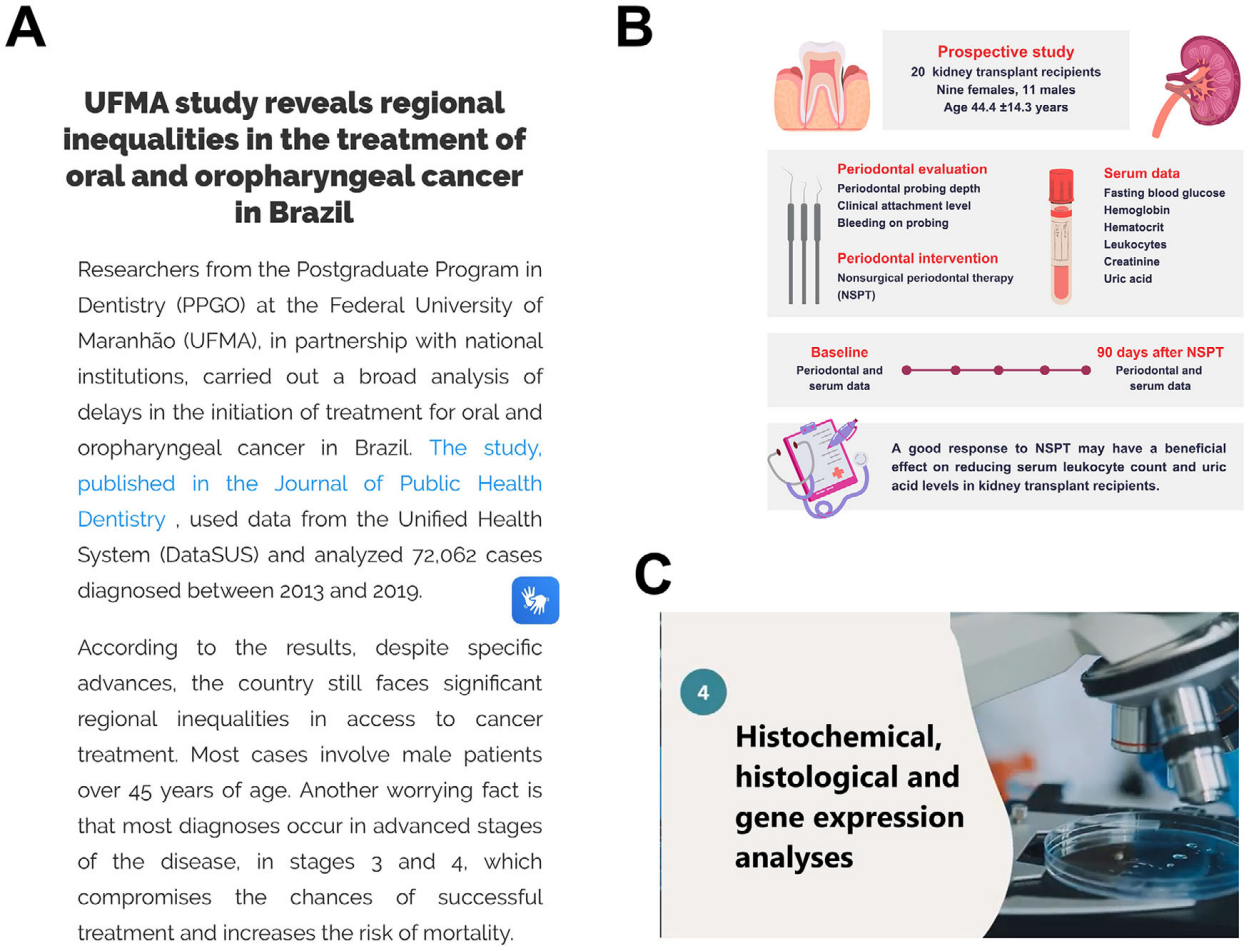
Examples of science communication outputs produced by students during the course: news articles published on the university's website (A), graphical abstracts (B), and video abstracts (C) designed for dissemination on social media platforms.

The course enabled students to develop a broad range of science communication skills, including writing news articles for the university's website, designing graphical abstracts, and producing video abstracts suitable for dissemination on platforms such as Instagram and YouTube (Figure 1).

The course emphasized experiential learning, encouraging students to transform their own research into multimedia content. Through the process of editing audiovisual materials, participants learned to simplify technical language without compromising scientific accuracy, making their research more accessible and relatable to the public. A key component of effective science communication addressed in the course was understanding the target audience. This knowledge is essential, as it influences the selection of language, tone, format, and dissemination channel. Different audiences have distinct levels of scientific literacy, interests, and informational needs, which must be considered when crafting outreach materials.

The course, with a total workload of 45 h, was offered once a year from 2022 to 2025. Over this period, 59 students completed the program, with 62.7% enrolled at the master's level and 37.3% at the doctoral level. All participants held a degree in Dentistry. Student perceptions of the course's contributions to the development of graphical abstracts, news articles, and video abstracts were evaluated using a post‐course questionnaire (Table 2). Response to the post‐course questionnaire was voluntary, and 33 graduates completed the survey, corresponding to 55.9% of the target population. A high proportion of students successfully applied the learned competencies after the course, with 66.7% creating graphical abstracts, 57.6% producing video abstracts, and 48.5% writing news articles. Most participants reported using these materials for academic purposes. Dissemination primarily occurred through personal and institutional social media channels. In addition, students identified knowledge democratization and combating misinformation as the main objectives of scientific dissemination in dentistry. Participants emphasized the need for improvements in content creation, audience‐oriented communication, and the practical application of research dissemination strategies. These findings align with the Commission on Dental Accreditation (CODA) Standard 2–17, which requires that students demonstrate communication and interpersonal skills necessary to interact effectively with diverse patient populations in multicultural work environments [6].

**TABLE 2:** Student perceptions regarding the contributions of the science communication course to the development of graphical abstracts, news articles, and video abstracts.

Questions	Response (*n *= 33)
After the course:	
Did you produce any graphical abstract?	Yes: 22/33 (66.7%)
Did you produce any news article?	Yes: 16/33 (48.5%)
Did you produce any video abstract?	Yes: 19/33 (57.6%)
For what purposes did you create these science communication materials?	
Academic purposes	Yes: 27/33 (81.8%)
Clinical advertising	Yes: 6/33 (18.2%)
Health guidance for patients	Yes: 5/33 (15.2%)
Which media outlets did you use to disseminate these materials?	
Personal social media	Yes: 11/33 (33.3%)
Institutional social media	Yes: 8/33 (24.2%)
Submission of an article to scientific journals	Yes: 11/33 (33.3%)
Scientific events	Yes: 7 /33 (21.2%)
What do you consider to be the main objectives of scientific dissemination in dentistry?	
Democratization of knowledge	Yes: 33/33 (100%)
Combating misinformation	Yes: 31/33 (93.9%)
Engaging society	Yes: 28/33 (84.8%)
Promotion of oral health	Yes: 26/33 (78.8%)
Encouraging research	Yes: 26/33 (78.8%)
Improving professional training	Yes: 23/33 (69.7%)
Training critical citizens	Yes: 22/33 (66.7%)
How did the activities in this course contribute to your training as a researcher and/or clinician? “quotes”	
“This course allowed me to understand the importance of good communication and appropriate language choice. It also taught me the relationship between the message and the target audience.”	
“It helped me improve my skills in creating graphical content.”	
“It makes scientific information easier to understand for doctors and the general public.”	
“It encouraged critical reading to identify the key points to be shared.”	

## 4 Lessons Learned

One of the key lessons learned in planning the course was the importance of balancing theoretical content with hands‐on activities. This approach ensured that students could immediately apply science communication strategies to their own research projects. Initially, many students were unfamiliar with audiovisual tools and scripting for non‐specialist audiences. However, these challenges diminished as they engaged in course activities and received additional support in using digital media resources.

The evaluation of student feedback highlighted the importance of incorporating pre‐ and post‐course assessments to more accurately measure changes in knowledge and practices. The absence of a pre‐course baseline in this initial offering is a limitation that will be addressed in future iterations to better measure the course's direct impact. Additional challenges included students’ limited familiarity with audiovisual production tools and a moderate post‐course survey response rate. These issues underscore the need for targeted pre‐course training and improved data collection strategies, such as implementing reminder schedules and setting aside dedicated in‐class time for evaluations. Planned improvements for future iterations include offering pre‐course workshops on basic digital media skills to enhance student preparation and optimize course efficiency.

By mastering these competencies, graduate students could enhance the visibility, accessibility, and societal impact of their scientific output. To support this progress, dental education must integrate dedicated courses and practical training in science outreach, empowering professionals to become active communicators of evidence‐based knowledge in society.

## Conflicts of Interest

The authors declare no conflicts of interest.
